# Supplementary material for: Resolving the full spectrum of human genome variation using Linked-Reads
Source: Genome Res. 2019 Apr;29(4):635–45. doi: 10.1101/gr.234443.118 (PMC6442396; doi:10.1101/gr.234443.118)
Supplement: Supplemental Material [file supp_gr.234443.118_Supplemental_longranger-2.2.zip › longranger-2.2.tar.bz2!/lr2.2-public/lib/stan/cmdstan/stan/doc/api/html/dense__e__metric_8hpp_source.html]

Stan: src/stan/mcmc/hmc/hamiltonians/dense\_e\_metric.hpp Source File


|  |  |
| --- | --- |
|  | Stan  2.5.0  probability, sampling & optimization |


- Main Page
- Related Pages
- Namespaces
- Classes
- Files

- File List
- File Members

All Classes Namespaces Files Functions Variables Typedefs Enumerations Enumerator Friends Macros Pages

- src
- stan
- mcmc
- hmc
- hamiltonians

dense\_e\_metric.hpp

Go to the documentation of this file.

1 #ifndef STAN\_\_MCMC\_\_DENSE\_\_E\_\_METRIC\_\_BETA

2 #define STAN\_\_MCMC\_\_DENSE\_\_E\_\_METRIC\_\_BETA

3

4 #include <boost/random/variate\_generator.hpp>

5 #include <boost/random/normal\_distribution.hpp>

6

7 #include <stan/math/matrix/Eigen.hpp>

8 #include <Eigen/Cholesky>

9

10 #include <stan/math/matrix/meta/index\_type.hpp>

11 #include <stan/mcmc/hmc/hamiltonians/base\_hamiltonian.hpp>

12 #include <stan/mcmc/hmc/hamiltonians/dense\_e\_point.hpp>

13

14 namespace stan {

15

16  namespace mcmc {

17

18  // Euclidean manifold with dense metric

19  template <typename M, typename BaseRNG>

20  class dense\_e\_metric: public base\_hamiltonian<M, dense\_e\_point, BaseRNG> {

21

22  public:

23

24  dense\_e\_metric(M& m, std::ostream\* e):

25  base\_hamiltonian<M, dense\_e\_point, BaseRNG>(m, e) {};

26  ~dense\_e\_metric() {};

27

28  double T(dense\_e\_point& z) {

29  return 0.5 \* z.p.transpose() \* z.mInv \* z.p;

30  }

31

32  double tau(dense\_e\_point& z) { return T(z); }

33  double phi(dense\_e\_point& z) { return this->V(z); }

34

35  const Eigen::VectorXd dtau\_dq(dense\_e\_point& z) {

36  return Eigen::VectorXd::Zero(this->model\_.num\_params\_r());

37  }

38

39  const Eigen::VectorXd dtau\_dp(dense\_e\_point& z) {

40  return z.mInv \* z.p;

41  }

42

43  const Eigen::VectorXd dphi\_dq(dense\_e\_point& z) {

44  return z.g;

45  }

46

47  void sample\_p(dense\_e\_point& z, BaseRNG& rng) {

48  typedef typename stan::math::index\_type<Eigen::VectorXd>::type idx\_t;

49  boost::variate\_generator<BaseRNG&, boost::normal\_distribution<> >

50  rand\_dense\_gaus(rng, boost::normal\_distribution<>());

51

52  Eigen::VectorXd u(z.p.size());

53

54  for (idx\_t i = 0; i < u.size(); ++i)

55  u(i) = rand\_dense\_gaus();

56

57  z.p = z.mInv.llt().matrixL().solve(u);

58

59  }

60

61  };

62

63  } // mcmc

64

65 } // stan

66

67

68 #endif

stan::mcmc::base\_hamiltonian< M, dense\_e\_point, BaseRNG >::model\_

M & model\_

**Definition:** base\_hamiltonian.hpp:59

stan::mcmc::dense\_e\_metric::dtau\_dp

const Eigen::VectorXd dtau\_dp(dense\_e\_point &z)

**Definition:** dense\_e\_metric.hpp:39

base\_hamiltonian.hpp

stan::mcmc::base\_hamiltonian< M, dense\_e\_point, BaseRNG >::V

double V(dense\_e\_point &z)

**Definition:** base\_hamiltonian.hpp:25

stan::mcmc::dense\_e\_metric

**Definition:** dense\_e\_metric.hpp:20

stan::mcmc::dense\_e\_metric::sample\_p

void sample\_p(dense\_e\_point &z, BaseRNG &rng)

**Definition:** dense\_e\_metric.hpp:47

stan::mcmc::dense\_e\_metric::dense\_e\_metric

dense\_e\_metric(M &m, std::ostream \*e)

**Definition:** dense\_e\_metric.hpp:24

stan::math::index\_type

Primary template class for the metaprogram to compute the index type of a container.

**Definition:** index\_type.hpp:21

stan::mcmc::ps\_point::p

Eigen::VectorXd p

**Definition:** ps\_point.hpp:48

stan::mcmc::dense\_e\_metric::~dense\_e\_metric

~dense\_e\_metric()

**Definition:** dense\_e\_metric.hpp:26

stan::mcmc::dense\_e\_metric::dphi\_dq

const Eigen::VectorXd dphi\_dq(dense\_e\_point &z)

**Definition:** dense\_e\_metric.hpp:43

stan::mcmc::dense\_e\_point::mInv

Eigen::MatrixXd mInv

**Definition:** dense\_e\_point.hpp:18

index\_type.hpp

dense\_e\_point.hpp

stan::mcmc::ps\_point::g

Eigen::VectorXd g

**Definition:** ps\_point.hpp:51

Eigen.hpp

stan::mcmc::dense\_e\_metric::tau

double tau(dense\_e\_point &z)

**Definition:** dense\_e\_metric.hpp:32

stan::mcmc::base\_hamiltonian

**Definition:** base\_hamiltonian.hpp:17

stan::math::e

double e()

Return the base of the natural logarithm.

**Definition:** constants.hpp:86

stan::mcmc::dense\_e\_point

**Definition:** dense\_e\_point.hpp:12

stan::mcmc::dense\_e\_metric::phi

double phi(dense\_e\_point &z)

**Definition:** dense\_e\_metric.hpp:33

stan::mcmc::dense\_e\_metric::dtau\_dq

const Eigen::VectorXd dtau\_dq(dense\_e\_point &z)

**Definition:** dense\_e\_metric.hpp:35

stan::mcmc::dense\_e\_metric::T

double T(dense\_e\_point &z)

**Definition:** dense\_e\_metric.hpp:28


---

[ Stan Home Page ]

*© 2011–2014,
Stan Development Team.*
